# A Web-Based Total Worker Health Intervention for Those Fighting Wildland Fires: Mixed Methods Development and Effectiveness Trial

**DOI:** 10.2196/47050

**Published:** 2023-10-25

**Authors:** Kerry Kuehl, Diane Elliot, Carol DeFrancesco, Wendy McGinnis, Susanna Ek, Bharti Garg

**Affiliations:** 1 Oregon Health & Science University Portland, OR United States

**Keywords:** wildland, firefighter, Total Worker Health, web-based, occupational safety, health promotion, wildland firefighter, web-based safety, mixed methods, occupational health, health and safety, health care worker, mobile phone

## Abstract

**Background:**

Fire seasons are longer, with more and larger wildfires, placing increased demands and risks on those fighting wildland fires. There are multiple agencies involved with fighting wildland fires and unique worksite conditions make meeting these workers’ needs a challenge.

**Objective:**

The aim of the study is to develop and establish the effectiveness of a web-based safety and health program for those fighting wildland fires.

**Methods:**

This mixed methods project had 3 phases. The initial qualitative phase assessed the needs of 150 diverse firefighters through interviews and focus groups across 11 US sites to establish and prioritize program content. Interview transcripts were read for thematic content with iterative readings used to identify, code, and rank health and safety issues. The second phase used that information to build a comprehensive Total Worker Health program for those fighting wildfires. The program content was based on the qualitative interview data and consisted of 6 core and 8 elective 30-minute, web-based modules primarily done individually on a smartphone or computer. The final, third phase evaluated the program with a quantitative prospective proof-of-concept, usability, and effectiveness trial among wildland firefighter participants. Effectiveness was assessed with paired 2-tailed *t* tests for pre- and post-Likert agreement scale survey items, adjusted for multiple comparisons. In addition to assessing mean and SD at baseline and postsurvey, observed effect sizes were calculated (Cohen *d*). Usability and reaction to the program among firefighters who responded to postsurvey were also assessed.

**Results:**

The qualitative themes and subthemes were used to inform the program’s content. For the effectiveness trial, 131 firefighters completed the presurvey, and 50 (38.2%) completed the postsurvey. The majority of the participants were White (n=123, 93.9%), male (n=117, 89.3%), with an average age of 41 (SD 12.9) years. Significant increases in knowledge and desired health and safety behaviors were found for both cancer (*P*<.001) and cardiovascular risk (*P*=.01), nutrition behaviors (*P*=.01), hydration or overheating (*P*=.001), binge drinking (*P*=.002), and getting medical checkups (*P*=.001). More than 80% (n=40) of postsurvey respondents agreed or strongly agreed that the program was easy to use and would recommend it to others.

**Conclusions:**

An innovative web-based safety and health promotion program for those fighting wildland fires was feasible, scalable, and usable. It improved the health and safety of those fighting wildland fires.

**Trial Registration:**

ClinicalTrials.gov NCT05753358; https://classic.clinicaltrials.gov/ct2/show/NCT05753358

## Introduction

In recent years, the size and severity of wildfires have increased. In the United States, the annual number of acres burned has doubled in the last 2 decades, breaking records in many states. In addition, more housing development near forests, an area referred to as the wildland-urban interface (WUI), creates an expanding aspect of wildfires [1-3]. These wildfire increases are straining the federal wildland firefighting workforce and local agencies responsible for nonfederal land wildfires. Federal legislation [4] and the Federal Emergency Management Agency, through its funding priorities, have recognized the critical need to support the well-being of those fighting wildland fires. We describe the development, assessment, and outcomes of a health and safety program designed for those fighting wildland fires.

Providing services to this dispersed and varied workforce is a challenge. The “typical” wildland firefighter is one of approximately 19,000 federal employees responding to wildfires that begin on federal lands. However, that number includes those working full-time year-round, full-time seasonal employees, and part-time seasonal workers. The Forest Service, within the US Department of Agriculture, employs nearly 70% of federal firefighters, with the remaining divided among 4 Department of the Interior agencies (Fish and Wildlife, Bureau of Indian Affairs, National Park Service, and Bureau of Land Management). The demographics, work histories, training, duties, and supervising hierarchy vary across all those agencies and types of workers.

The term wildland fire refers not only to federal land forest fires but any nonstructure fire that occurs in vegetation and natural fuels in a forest, grassland, brushland, or land sown to crops. Accordingly, nonfederal firefighters, through city, county, or district fire agencies, are responsible for responding to wildfires that begin on state, local, and private lands. A large percentage of the more than 400,000 career structural firefighters are involved in both fighting WUI fires and deployed to fire camps during the peak of fire seasons. Finally, wildland firefighting also includes the largest group of US firefighters, the 800,000 volunteers who comprise the majority of those protecting smaller rural communities. These individuals often are those first arriving at a wildland fire. Each of these sectors, often further regionally subdivided, has unique training and certification processes.

Despite these complexities, the need is great to address the safety and health of those fighting wildland fires. There is a growing literature on the safety and health risks associated with fighting wildland fires. Smoke exposure is an obvious aspect, as unlike structural firefighters, they cannot use self-contained breathing protection and are exposed to smoke’s particulate matter and other toxic or carcinogenic contaminants. That exposure is associated with an elevated risk of respiratory and cardiovascular disease [5]. In 2017, Domitrovich et al [6] published a comprehensive review of wildland firefighters’ respiratory exposures and concluded that their “risk for lung cancer mortality increases nearly linearly with exposures over time.” Moreover, assays for oxidative stress, proximal markers for heightened cancer risk, have found a significant increase in DNA damage among wildland firefighters [7].

Those fighting wildfires have an increased risk for cardiovascular disease. Despite public perceptions to the contrary, firefighters have a concentration of the most prevalent harmful health behaviors, such as unhealthy diets, lack of sleep, and other cardiovascular risk factors [8,9]. Those fighting wildland fires, especially older individuals involved in incident command, have a heightened risk for elevated cholesterol, being overweight, and hypertension [9]. A survey of former wildland firefighters found that the total work years significantly correlated with greater odds of having both hypertension and heart disease [10,11]. Demanding physical work, exposure to heat, and injuries are an accepted aspect of wildland firefighting. In general, firefighters have 4 to 8 times the rate of work-related injuries compared to other comparable industries [12]. A retrospective examination of wildland injuries indicated that slips, trips, and falls were the most common injuries, and injury severity increased later in the fire season [12]. Conditions in fire camps also make sleep a challenge. Suboptimal sleep was observed among wildland firefighters both while on the line and during nonfire work so that approximately half of the sleep measures were outside the adult optimal range [13].

A recent *Atlantic* magazine article, “Quiet Rise in Wildland-Firefighter Suicides,” focused attention on the growing stress that accompanies fighting wildland fires [14]. These workers experience family disruptions during weeks on the fire line as well as life-threatening physical hazards and traumatic events as they interface with loss of property and displaced individuals [15]. These factors occur in a male-dominated profession, where seeking mental health care may be stigmatized. Recent federal legislation has been passed to specifically address the mental health needs of wildland firefighters [16].

The literature on wildland firefighting confirms that this work adversely affects mental health, physical well-being, and social connections. A recent 10-person panel of wildland firefighting experts ranked the needs of wildland firefighters, and the highest ranking was for a comprehensive, holistic program called Total Worker Health (TWH) [17]. The name Total Worker Health, trademarked by the US Centers for Disease Control and Prevention, refers to the combination of policies, programs, and practices that integrate protection from work-related health hazards with promotion of illness prevention [18]. As founding members of the National Institute for Occupational Safety and Health–funded Oregon Healthy Workforce Center, we have been at the forefront of TWH efforts and have developed programs that positively impacted first responder groups and other workers [19-22].

Involving workers in a structured process defining their safety and health needs is a key tenet of TWH [23]. Accordingly, a foundational assumption was that meaningful engagement and empowerment of front-line firefighters were essential in establishing the scope of the proposed program. In addition to the face validity of participatory data gathering from future end users, this process can also increase subsequent program engagement and commitment [24-26]. Along with a TWH model and participatory approach, we drew on our evidence-based behavior change model, which we have successfully used to advance the health and safety of other occupational groups [27]. Our experience with creating successful occupational health and safety behavior change programs includes more than 2 decades of research with structural firefighters and establishing the only evidence-based TWH program tailored to the needs of career structural firefighters [21,22,28,29]. Our objective was to provide a comprehensive program that included safety, health, and organizational topics to promote TWH among those fighting wildland fires.

## Methods

### Overall Study Design

This mixed methods TWH for wildland firefighters project had 3 phases. The initial qualitative phase was assessing the needs of wildland firefighters across segments and geographic locations to identify and prioritize program components. During that first phase, we recruited firefighters plus collected and analyzed focus group and interview data to assess their needs. The second phase was to use the phase 1 findings to build a comprehensive, engaging TWH program for those fighting wildfires. It needed to be accessible on smartphone, tablet, or computer and suitable for individual, group, and classroom settings. The final, third phase was to evaluate that program with a prospective single-arm proof-of-concept, usability, and effectiveness trial among all types of wildland firefighters in different geographic locations.

### Phase 1: Qualitative Needs Assessment

#### Site and Participant Recruitment

The sites for both the initial qualitative needs assessment and the later effectiveness trial are listed in Table 1. We chose sites to be geographically diverse and to include representatives from all types of individuals fighting wildland fires—federally funded firefighters (full-time, full-time seasonal, and seasonal only), as well as career structural and volunteer firefighters with fire camp and WUI experience. For example, the Northwest region site 1 is a large urban department of career structural firefighters, all of whom are also certified in wildland fire suppression. Northwest sites 2 and 3 are smaller districts, and all individuals have wildland experience and include career structural firefighters, career Forest Service employees, and volunteers.

At each location, we contacted the top individuals in management as well as union representatives and health or safety officers if available. This was followed by a conference call or in-person meeting to explain the study. With agreement to proceed, information concerning the study was disseminated widely to all eligible firefighters. We arranged for a convenient time to meet with firefighters, for example, at an existing training session or during another prescheduled meeting to explain the study and obtain informed consent prior to any data gathering. Participants in this study were a convenience sample of firefighters able to attend informational sessions on the study, although we did make additional efforts to involve women and ethnic minority firefighters.

**Table 1:** Participating sites and number or type of firefighters in study phases 1 and 3.

Location and type of department or district			Focus groups, n (participants, n)		Presurvey participants, n		Pre- and postsurvey participants, n
**Region 6—Northwest**							
	Large urban department (site 1)	2 (4 S^a^)		4 S		2 S	
	Small rural fire department (site 2)	3 (3 S, 10 V^b^)		13 V, 5 S, 1 DNI^c^		8 V, 5 S	
	Small rural district (site 3)	4 (9 S, 3 V)		7 V, 3 S		1 V, 3 S	
**Region 4—Intermountain**							
	Medium federal district (site 4)	3 (3 FS^d^)		22 FS		5 FS	
	Large urban fire department (site 5)	4 (9 S)		1 S		0	
**Region 1—Northern**							
	Small rural fire department (site 6)	4 (11 S, 7 V)		13 V, 11 S		6 V, 3 S	
	Small rural fire department (site 7)	2 (3 S, 3 V)		8 S, 7 V		0	
**Region 8—Southern**							
	Large urban or rural fire district (site 8)	11 (81 S, 2 V)		11 S		5 S	
**Region 9—Eastern**							
	Medium urban or suburban department (site 9)	N/A^e^		38 S		23 S	
National Interagency Fire Center, Women in Fire organization			2 (2 FS)		6 FS		3 FS
Total			35 (25 V, 120 S, 5 FS)		40 V, 81 S, 28 FS, 1 DNI		15 V, 41 S, 8 FS

^a^S: career structural with wildland-urban interface experience.

^b^V: volunteer.

^c^DNI: did not identify.

^d^FS: Forest Service or other state or federal agency employee.

^e^N/A: not applicable.

#### Ethics Approval

The Oregon Health & Science University Institutional Review Board approved the study and its procedures (21022).

#### Qualitative Data Collection

We had planned to visit sites to collect the focus group data in person. However, we had just completed meeting with 2 local sites when COVID-19 travel restrictions made in-person visits impossible. Our methods were altered to assess participants using web-based Webex methods. The validity of these methods for data gathering has been reported [30,31]. We collected qualitative data from all participating sites.

Prior to the focus groups, each participant signed informed consent, followed by a brief confidential survey. The survey oriented them to the domains included in the interviews, and they were asked to identify the importance of 13 health topics (1=strongly disagree to 5=strongly agree) and list their top 3 most relevant topics.

Survey completion was followed by group interviews for firefighters and individual key-informant interviews for those in charge, with each lasting approximately 60 minutes. The individual and group interviews use a trained facilitator and semistructured interview guide (Multimedia Appendix 1) and a scribe to track participant comments and take notes. KK, CD, and SE performed these tasks. Open-ended questions with follow-up probes explored the safety and health-content domains, their perceived needs, prior experiences with safety and wellness efforts, and unique concerns when fighting wildland fires. Interviews were audiotaped, transcribed verbatim, reviewed for accuracy, redacted names, and assembled for analysis.

#### Qualitative Analysis

For the qualitative analyses, 4 members of the research team read 4 different transcripts to inductively identify an initial list of codes, which were added to the list of topics from the survey. All these proposed codes were collapsed into 1 list and loaded into Kardsort [32], an open-source, cloud-based software that allows researchers to sort “cards” into different categories. For this purpose, categories for codes included “keep,” “modify,” “too narrow,” “too broad,” and “do not include.” In total, 4 members of the research team sorted all of the codes into these categories, with results exported to a spreadsheet. R (R Foundation for Statistical Computing) was used to organize the codes by number of “votes,” combine similar codes, and remove codes not considered to be useful.

The interview and focus group transcripts were loaded into Taguette [33], an open-source, cloud-based, qualitative software. Each transcript was assigned a primary and a secondary coder. The coding was used to identify themes with the largest number of references in the transcripts. For each theme, we identified any subthemes and pulled representative quotes for each theme and subtheme. Quality control to protect validity and avoid bias involved iteratively discussing and reconciling themes and subthemes until consensus across the team was reached. The research team also used the COREQ (Consolidated Criteria for Reporting Qualitative Research) checklist (Multimedia Appendix 2) for properly reporting qualitative research.

## Results

### Qualitative Results

Data were collected from February 2020 through November 2020. We held 24 focus groups (5 in-person and 19 Webex) that ranged from 2 to 15 firefighters with an average of 6 (SD 3.04) participants per group. In addition, we held 11 key informant interviews of supervisors (2 in-person and 9 Webex). These occurred at 10 locations and are presented in Table 1. In total, 150 participated in focus groups and interviews. Their average age was 41 (SD 11.6) years with a mean of 12.5 (SD 10.3) years fighting wildland fires. In total, 93% (n=140) were male, and 93% (n=140) were White. In total, 17% (n=25) were volunteer firefighters, while the remainder were Forest Service or career structural firefighters.

All the survey health and safety topics (hydration or heat injury, physical fitness, personal protective equipment, cancer risk, cardiovascular disease, mental health, sleep or fatigue, work-life balance, nutrition, and injury prevention) were rated highly by those fighting wildland fires with no significant differences among the topics. When asked for their top 3 topics, more than 40% included physical fitness, hydration, personal protective equipment, and mental health.

Table 2 presents a listing of the topics and subthemes from the qualitative analyses with the number of utterances associated with each theme. Transcript quotes related to each theme and subtheme were iteratively read by the group to inform the program content. Examples of that are presented for selected topics.

The importance of hydration was often mentioned. Information concerning those subthemes was used in constructing the hydration module. Subthemes for hydration and heat included the relationship to nutrition and the importance of drinking and eating before you thought you needed it, even before deployed, “you know, had a couple of cups of coffee in the morning. Wasn't well hydrated got out there and spent an hour and a half on hillside and a hundred-degree temperatures and I ended up barely making it to the fire station.” Also noted were regional variability in heat and humidity that may place a firefighter deployed to a different region particularly at risk. “You get people from parts of the country that are closer to sea level, more humidity. They're not used to the environment.” Many different types of replacement fluids were mentioned (eg, Propel, Gatorade, Emergen-c, and DripDrop), resulting in clear general recommendations being included in the program. Although many were familiar with the term rhabdomyolysis, few knew the risks particularly related to vigorous preseason training when not in shape. As a result, that information was accompanied by a link for watching the US Department of Agriculture YouTube informational video on rhabdomyolysis among wildland firefighters.

As demonstrated by the number of tagged utterances in Table 2, a striking finding was the absence of concerns about heart disease. Although a few firefighters acknowledged that risk, when probes about cardiovascular issues were used, it often resulted in a discussion about cardio or endurance training. Similarly, most reported avoiding physician preventative visits: “the only time I get a physical is when I am forced to.” Accordingly, the cardiovascular module included information about the heightened risk of heart disease among firefighters and modifiable risk factors. The medical checkup module made the analogy of equipment maintenance and the components of career structural firefighters’ National Fire Protection Agency 1582 physical [34] and how wildland firefighters’ occupational risk information could be shared with their primary care providers.

Nutrition was a theme that was integrated with physical health and the logistics of what they could and could not control. Nutritional demands mentioned at fire camps were so unique that program content was partitioned as nutrition on the line and off the line. For nutrition on the line, guidance focused on matching intake to caloric demands and eating frequently, like an endurance athlete. The firefighters told us how they wanted the information presented: “Streamline information source for people to kind of like, you know, this will help fuel you in a correct way versus, you know, like I said, this, the sugar bombs that destroy you all day long.” The comments relevant to a topic were reviewed as that module was built. Those “vicarious experiences” provided by the focus groups provided a deeper understanding and “tacit knowledge” more influential than “propositional knowledge” for informing the program’s content [35,36]. In addition, it allowed punctuating the program content with relevant firefighter quotes for emphasis.

**Table 2:** Qualitative themes and subthemes.

Themes (number of times coded in transcripts)	Subthemes
Nutrition (152)	Logistics Quality of food or choices Integration with other themes Enough food
Injuries or fatalities (69)	Fatigue Distractions Physical fitness Training, rules, and policies Wrong equipment
Heart disease prevention or physical (48)	No subthemes
Mental health (260)	Support Communicate Coping mechanisms Fatigue Changing culture
Stress relief with substance use, alcohol, tobacco, and caffeine (32)	Coping mechanism Off-season or seasonal Cultural toughness Age-related, younger firefighters Responsible use Leadership impact on employment Caffeine
Illnesses, being sick (21)	Camp crud COVID-19 Smoke inhalation Heat exhaustion Vascular Cancer
Hygiene (39)	Facilities or logistics Time Rules or policy Cultural toughness Gear Health hazards Women COVID-19 Wildland versus structural
Sleep and fatigue (207)	Environment or infrastructure Work-life balance Poor decision-making Physical fitness Work to rest ratio Policies or schedules Leadership Volunteers Mental health
Hydration and heat (117)	Nutrition Personal protection or replacement fluids Fatigue Illness or rhabdomyolysis Conditions
Leadership (70)	Support What works, practical tips, curriculum, and program suggestions Communicate
Physical fitness (245)	General importance Barriers Different types of firefighters Specific type of exercise

### Phase 2: Build the Program

The program was built based on the needs assessment, consultation with content experts, and review of available wildland training. Translating that information into a scope, sequence, and educational process was aided by our long experience constructing evidence-based safety and health curricula for varied occupational groups, including the fire service and other first responders [19-22].

The program is structured as 30-minute modules that can be done individually, with a partner, as a group, or in a classroom setting. The 6 core modules are supplemented with 8 elective modules (Textbox 1). The program can be accessed on a smartphone, tablet, or computer, and if needed, can be downloaded as a PDF. Participants had access to the program, and they were free to do the modules at their own pace, including repeating content. The suggested format was to do 1 module a week, and after doing the core modules, do at least 4 of the elective modules.

The sessions were built using Articulate 360 [37], which is a well-established subscription platform for e-learning course development. It includes access to authoring apps and a library of course assets. For example, it has hundreds of potential dynamic components, such as interactive games, quizzes, reveals, and engaging multipath scenarios. It provides a consistent learner experience across all devices (computer, smartphone, or tablet).

Each of the modules contains approximately 20 wireframes. The wireframes are the baseline structure to which interactive features (eg, games, quizzes, reveals, matching, videos, and links to other content) can be attached. Each module follows a similar format. New knowledge is actively processed and placed in context with the learner’s own experiences, such as by completing quizzes, watching a 2-minute video, or reflecting on experiences. Each content area concludes by asking individuals to personalize specific behavioral objectives. In addition, the use of phase 1’s qualitative findings to build a program designed by wildland firefighters for wildland firefighters was emphasized with deidentified firefighter quotes used to underscore content in each module. Modules 2-6 begin with a follow-up on the prior session’s respective objectives to enhance self-efficacy for positive change, reinforce intrinsic motivation, and if done as a group, social support. This structure has been repeatedly shown effective, and we have demonstrated the validity of this framework for effective behavior change [27].

**Figure 1:**
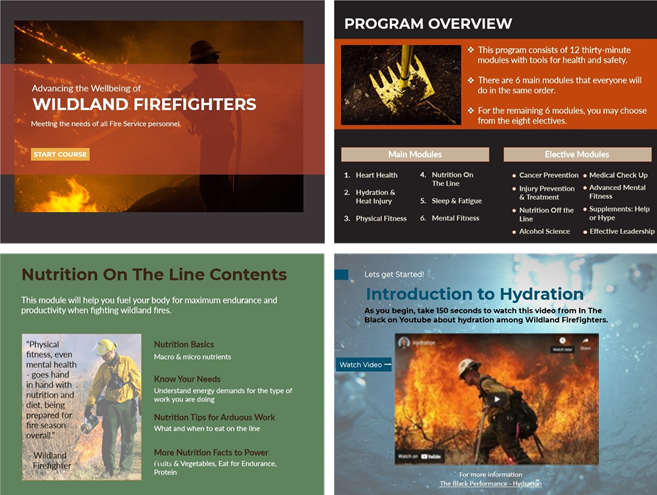
Selected wireframes from the program.

Figure 1 demonstrates single “wireframes” from more than 20 web-based wireframes per module. The selected wireframes may appear as a static series of slides, but each has dynamic features not depicted in these pictures that make it engaging and importantly demand processing the material to personalize it, enhance retention, and facilitate future positive actions. Once completed, the program was accessible 24-7 from a study-hosted website.

**Program modules.**

**Core modules**

Core 1 Heart health

Core 2 Hydration and heat injury

Core 3 Physical fitness

Core 4 Nutrition on the line

Core 5 Sleep and fatigue

Core 6 Mental fitness

**Elective modules**

Elective advanced mental fitness

Elective alcohol science

Elective cancer prevention

Elective injury prevention and treatment

Elective medical checkup

Elective nutrition off the line

Elective effective leadership

Elective supplements: help or hype

### Phase 3: Quantitative Program Outcomes

#### Participant Recruitment

Informational emails about the final phase or effectiveness trial of the program were sent to management at each of the original sites and distributed to those firefighters. In addition, contact information obtained during phase 1 was used to reconnect with individuals and invite them to participate in phase 3. At a site, those who did not participate in phase 1 but expressed interest in the program were allowed to participate in phase 3. Web-based meetings were set up for interested firefighters at sites 1, 8, and 9. In-person visits were made to sites 2 through 7. During those web-based and in-person meetings, researchers answered questions, explained and obtained consent, administered the baseline preprogram survey, and led participants through the first program module. Not all participants from phase 1 were still at participating sites, and so, those who no longer were at the participating sites did not complete the program.

#### Quantitative Data Collection

A quasi-experimental single group pre- to postdesign was used to evaluate the program. The research team’s experience with phase 1 and the practical issues of data collection among busy firefighters being contacted digitally led to the use of a brief survey in phase 2 rather than the longer instruments with validated constructs used in our prior research [19-22]. In addition, the survey included information about usability and postparticipation impressions of the program using a 7-point Likert agreement scale (1=strongly disagree to 7=strongly agree).

The link to the follow-up survey was sent to a participant’s email approximately 4 months after they received the 14-week program. Those not responding to an initial email were contacted by 2 additional emails and a personal phone call asking them to complete the survey. A technical issue at site 2 precluded using its data.

#### Quantitative Analyses

Demographics and other characteristics of all the included participants were summarized using mean (SD) or median (IQR) for continuous variables and frequency (percentage) for categorical variables. To assist in assessing whether there was a selection bias among those completing the postsurvey, we compared all firefighters who completed postsurvey to those not completing postsurvey using chi-squared or Fisher exact test for categorical variables and independent 2-sample 2-tailed *t* tests for continuous variables [38]. Presurvey and postsurvey were compared using paired 2-tailed *t* tests to assess within-group changes. In addition to assessing mean and SD at baseline and postsurvey, we also looked at observed effect sizes (Cohen *d*). Calculation of Cohen *d* was done by dividing the mean difference (subtracting postsurvey mean from baseline) by the average SD of both repeated measures (ignoring the correlation between repeated measures) [39]. Based on the benchmarks suggested by Cohen, effect sizes are referred as small (*d*=0.2), medium (*d*=0.5), and large (*d*=0.8) [40]. Usability and reaction to the program among firefighters who responded to postsurvey were assessed using mean and SD for continuous variables and frequency (percentage) for categorical variables. Because of the multiple comparisons, statistical significance was set at 0.01 so as to decrease type-1 error [41]. Analyses were done using Stata (version 17; Stata Corp).

#### Quantitative Results

A total of 150 firefighters completed the prestudy and had access to the program. We excluded firefighters from site 2 (n=19) due to technical issues. Among the 131 firefighters completing the presurvey, 50 (38.2%) completed the postsurvey. The pre or postparticipants included individuals from all geographic areas. The completion rate was comparable among sites. As shown in Table 3, there were no meaningful demographic differences between those completing and not completing the postsurvey, except that the firefighters not completing the postsurvey were younger as compared to firefighters who completed postsurvey (mean 39.1, SD 12.9 vs mean 44.4, SD 12.3; *P*=.02).

Table 4 shows that there was a significant increase in knowledge of blood pressure (mean change 0.54, 95% CI 0.23-0.85; *d*=0.49), cardiovascular risk factors (mean change 0.68, 95% CI 0.22-1.15; *d*=0.43), and cancer risk factors (mean change 0.76, 95% CI 0.34-1.16; *d*=0.52) after the survey. In addition, their intention to get a physical examination (mean change 0.76, 95% CI 0.32-1.20; *d*=0.49) and commitment to seeing a primary care provider increased by a modest effect size (mean change 0.58, 95% CI 0.20-0.96; *d*=0.44). Intention to eat every 2 hours (mean change 0.72, 95% CI 0.25-1.18; *d*=0.44), knowledge about protein-containing foods (mean change 0.86, 95% CI 0.32-1.40; *d*=0.45), and knowledge of binge drinking increased by modest effect size (mean change 0.51, 95% CI 0.18-0.83; *d*=0.45). There was no statistically significant difference in physical activity as the firefighters were already getting 150 minutes of physical activity at baseline. Most did the modules alone (n=40, 88%), and most used a computer or tablet (n=42, 84%) with only 14% (n=7) using their smartphone.

Usability has several dimensions, including user satisfaction, effectiveness, efficiency, and flexibility in use [42]. Based on self-report, 80% (n=40) agreed or strongly agreed that the program was easy to use, and 82% (n=41) felt that the program should be part of their training.

**Table 3:** Comparison of those completing and not completing the postsurvey. The chi-squared test, Fisher exact test, or independent 2-sample 2-tailed *t* test were used.

			Total (n=131)		Completed pre- and postsurvey (n=50)		Completed only presurvey (n=81)		*P* value	
**Race or ethnicity, n (%)**										.48
	Non-Hispanic White	123 (93.9)		47 (94)		76 (94)				
	Non-Hispanic Black	1 (0.8)		1 (2)		0 (0)				
	Hispanic	3 (2.3)		1 (2)		2 (2)				
	American Indian and Alaska Native	1 (0.8)		1 (2)		0 (0)				
	Other	2 (1.5)		0 (0)		2 (2)				
	Unknown	1 (0.8)		0 (0)		1 (1)				
Age (years), mean (SD)			41.1 (12.9)		44.4 (12.3)		39.1 (12.9)		.02	
BMI (kg/m^2^), mean (SD)			27.3 (4.1)		27.9 (3.8)		26.9 (4.3)		.19	
**Gender, n (%)**										.52
	Male	117 (89.3)		45 (90)		72 (89)				
	Female	13 (9.9)		4 (8)		9 (11)				
	Not reported	1 (0.8)		1 (2)		0 (0)				
Time worked on wildland fires (years), mean (SD)			8.5 (9.7)		7.3 (8.7)		9.2 (10.2)		.30	
**Current position, n (%)**										.38
	Volunteer firefighter	27 (20.8)		8 (16)		19 (24)				
	Career firefighter	103 (79.2)		42 (84)		61 (76)				

**Table 4:** Quantitative program evaluation (phase 3) pre- and postsurvey results (1=strongly disagree to 7=strongly agree).

	Presurvey, mean (SD)	Postsurvey, mean (SD)	Mean change (95% CI)	Effect size (Cohen *d*)	*P* value^a^
I know my risk factors for cancer	5.31 (1.57)	6.06 (0.83)	0.76 (0.34 to 1.16)	0.52	<.001
I know my blood pressure	5.56 (1.47)	6.10 (1.07)	0.54 (0.23 to 0.85)	0.49	.001
I intend to get a physical examination once a year	5.44 (1.86)	6.20 (0.88)	0.76 (0.32 to 1.20)	0.49	.001
When feeling overheated, the first thing to do is stop working	4.04 (1.69)	5.06 (1.61)	1.02 (0.41 to 1.62)	0.48	.001
I know the definition of binge drinking	5.95 (1.15)	6.47 (0.61)	0.51 (0.18 to 0.83)	0.45	.002
Protein is contained in many foods, including bread and rice	3.66 (1.93)	4.52 (1.99)	0.86 (0.32 to 1.40)	0.45	.002
I intend to eat every 2 hours when doing arduous wildland firefighting	4.06 (1.41)	4.78 (1.51)	0.72 (0.25 to 1.18)	0.44	.003
It is important for me to have a primary care provider	5.58 (1.48)	6.16 (0.99)	0.58 (0.20 to 0.96)	0.44	.003
I know my risk factors for cardiovascular disease	5.47 (1.42)	6.16 (1.02)	0.68 (0.22 to 1.15)	0.43	.01
Getting less than 5 hours of sleep lowers testosterone levels equal to someone 10 years older	5.36 (1.01)	5.84 (0.91)	0.48 (0.16 to 0.79)	0.43	.004
Carbohydrates are the primary fuel for moderate to intense physical activity	5.22 (1.18)	5.64 (0.87)	0.42 (0.09 to 0.74)	0.37	.01
I use alcohol to get to sleep	2.12 (1.51)	1.70 (1.16)	–0.42 (–0.79 to –0.04)	0.32	.03
I intend to keep track of my risks for cardiovascular disease	5.20 (1.41)	5.59 (1.32)	0.39 (0.03 to 0.74)	0.31	.03
Power naps will restore alertness and reduce accidents	5.44 (1.37)	5.78 (1.29)	0.34 (0.02 to 0.66)	0.29	.04
I can get in shape 2 weeks before a wildland fire season	2.53 (1.57)	2.04 (1.13)	–0.48 (–0.96 to –0.01)	0.29	.04
Dehydration begins to affect performance when fluid loss equals 2% of body weight	5.24 (1.15)	5.64 (1.19)	0.40 (–0.02 to 0.82)	0.27	.06
Drinking alcohol increases my cancer risk	5.26 (1.22)	5.52 (0.99)	0.26 (–0.10 to 0.62)	0.20	.15
Staying awake for 24 hours is equivalent to blood alcohol of 0.08, the legal limit	5.74 (1.03)	5.94 (1.04)	0.20 (–0.12 to 0.52)	0.17	.22
I am able to bounce back from stressful events	5.82 (0.92)	5.96 (0.73)	0.14 (–0.10 to 0.38)	0.16	.25
I need supplements to balance additional nutritional needs for being a wildland firefighter	4.10 (1.54)	4.38 (1.52)	0.28 (–0.26 to 0.82)	0.15	.30
In general, I manage stress in a healthy way	5.28 (1.07)	5.40 (0.95)	0.12 (–0.17 to 0.41)	0.12	.41
On steep and rocky terrain, it is best to move quickly to reduce the risk of injury	1.89 (0.89)	2.00 (0.91)	0.10 (–0.18 to 0.38)	0.10	.47
Before going to sleep, I wipe soot and ash from my skin	4.58 (1.97)	4.76 (2.18)	0.18 (–0.04 to 0.80)	0.08	.56
I get 150 minutes of moderate physical activity per week (including work)	5.48 (1.74)	5.58 (1.51)	0.10 (–0.36 to 0.57)	0.06	.67
I feel overwhelmed with my work	3.16 (1.52)	3.10 (1.61)	–0.06 (–0.53 to +0.41)	0.04	.79
I am currently trying to moderate my drinking	3.76 (2.01)	3.84 (2.07)	0.08 (–0.57 to 0.73)	0.03	.81
I can rely on people at work to support me	5.36 (1.37)	5.32 (1.36)	–0.04 (–0.46 to 0.37)	0.03	.85
During the last month, I felt significantly depressed	2.60 (1.59)	2.58 (1.48)	–0.02 (–0.35 to 0.31)	0.02	.90
Camelback systems are more effective in maintaining hydration than canteens	4.47 (1.26)	4.47 (1.56)	0.00 (–0.46 to 0.46)	0.00	>.99

^a^Paired 2-tailed *t* test.

## Discussion

### Principal Results

A TWH program integrating health promotion with health protection was feasible, scalable, and positively impacted the health and safety outcomes of those fighting wildland fires. TWH involves integrating health and safety with organizational and environmental policy. This program integrated safety and health into the topics presented, for example, leadership module that included supervisory training, and included the organizational training policies of the wildland firefighter departments.

Recent studies document the adverse impacts of wildland firefighting on mental and physical health [43,44]. Those concerns include heightened cardiovascular risk factors [44], and they are exposed to a multitude of carcinogenic substances primarily through respiratory and dermal exposure [45,46]. A significant finding was that following the program, participants better knew their risks for heart disease and cancer, as well as their blood pressure. Importantly, they had greater intentions to get a physical examination following the program. The program’s first module was on cardiovascular disease, focusing on the modifiable risk factors. One of the elective sessions, called “medical checkup,” emphasized the importance of having a primary care provider and getting an annual physical and laboratory assessment. Structural firefighters have a mandatory annual comprehensive medical examination and laboratory studies dictated by the National Fire Protection Act 1582 [34] that includes screening for cardiovascular disease and malignancies. Wildland firefighters do not have a uniform standard of care for annual medical assessments, and the elective module suggested ways that wildland firefighters might inform their medical providers about their occupational health risks.

Significant dietary changes were observed in several important areas. Participants reported a significant increase in their intentions to eat frequently when doing strenuous work. Participants also better understood that carbohydrates are the preferred fuel for high-intensity work and that protein is in plant-based foods. Those changes are relevant as it is well established that supplemental feedings during exercise lasting more than 2 hours result in increased ability to complete physical work [47]. Tracking dietary intake in fire camps indicated firefighters often ate foods higher in dietary fat and protein rather than the carbohydrates needed for their arduous work [48]. Despite the recognized importance of hydration and heat injury during wildland firefighting, program participants still significantly increased their understanding of appropriate actions when feeling overheated.

### Comparison With Prior Work

Wildland firefighters are a challenging group to study. Most of the published literature focuses on the short-term health effects of working a fire season on injuries, respiratory effects, skin exposure, hearing impairment, and mental health issues [43]. Koopmans et al [43] highlighted 5 prevention or mitigation studies, but the limitations of these reports included simulated wildland fire settings and small sample sizes. In addition, they generally focused on one-dimensional interventions, such as personal protective equipment to reduce respiratory exposure and hearing protection and improved hygiene to reduce dermal carcinogen exposure. We and others have reported on more comprehensive programs for career structural firefighters [21,22,49,50]. To our knowledge, this is the first report of a TWH program for those fighting wildland fires.

### Limitations

We recognize the limitations of a single-arm pre- to poststudy. However, participants were selected to be representative of a spectrum of wildland firefighters and geographic areas to enhance generalizability. Although the pretest may sensitize individuals, we did not find significant changes in all areas, suggesting sensitization and social desirability accounts for all the changes.

A second limitation or challenge of this study was this research trial started prior to the COVID-19 pandemic with the Oregon fire department’s qualitative assessment being conducted and completed by in-person research staff. We had planned to visit all sites to collect the focus group data in person. Once the pandemic started in March 2020, the strict COVID-19 travel restrictions made in-person visits impossible. We altered our methods to assess participants using web-based Webex methods. The validity of these methods for data gathering has been reported [30].

A third challenge occurred during the postintervention quantitative assessment. Our plan was to collect data near the end of the wildfire season, at which time the majority of wildland firefighters would have returned to their department from deployment. Unfortunately, our postintervention data collection occurred during the largest and most devastating wildfires in US history. It was tragic to hear that one of our Oregon study fire departments had a wildland firefighter fatality, and some of our study participants either lost personally or knew someone who had lost their homes and buildings. In a study fire department in Montana, one of our study participants suffered a tragic accident with severe burns over most of his body during a wildfire, having to be airlifted to a large urban hospital burn unit. Our postintervention study subject numbers reflected this prolonged and devastating fire season that took its toll on firefighters.

TWH includes individual and organizational strategies integrating protection from work-related safety and health hazards to advance worker well-being, including injury and illness prevention. A limitation of this program focused only on the individual level and did not address the organizational structure and policy.

### Conclusions

The potential benefits of this novel web-based program targeting a high-risk group will advance the health and safety of those fighting wildland fires at minimal expense (the program is free). As designed and developed, the platform and structure can easily be adapted for all fire service agencies as part of a classroom training setting or incorporated into an educational curriculum learning management system to be made accessible and free to all firefighters and agencies.

## Appendix

Multimedia Appendix 1 Federal Emergency Management Agency Wildland Firefighter focus group or individual interview guide.

Multimedia Appendix 2 COREQ (Consolidated Criteria for Reporting Qualitative Research) checklist.

## Abbreviations

COREQ: Consolidated Criteria for Reporting Qualitative Research
TWH: Total Worker Health
WUI: wildland-urban interface

## References

[1] (2021). Wildfire statistics. Congressional Research Service.

[2] (2015). The rising cost of wildfire operations: effects on the Forest Service's non-fire work. United States Department of Agriculture.

[3] (2022). Wildland fire: barriers to recruitment and retention of federal wildland firefighters. United States Government Accountability Office.

[4] (2021). H.R.3684—infrastructure investment and jobs act. US Congress.

[5] Brook RD, Rajagopalan S, Pope CA, Brook JR, Bhatnagar A, Diez-Roux AV, Holguin F, Hong Y, Luepker RV, Mittleman MA, Peters A, Siscovick D, Smith SC, Whitsel L, Kaufman JD, American Heart Association Council on Epidemiology and Prevention, Council on the Kidney in Cardiovascular Disease, and Council on Nutrition, Physical Activity and Metabolism (2010). Particulate matter air pollution and cardiovascular disease: an update to the scientific statement from the American Heart Association. Circulation.

[6] Domitrovich JW, Broyles GA, Ottmar RD, Reinhardt TE, Naeher LP, Kleinman MT, Navarro KM, Mackay CE, Adetona O (2017). Wildland fire smoke health effects on wildland firefighters and the public. Joint Fire Science Program.

[7] Fritschi L, Glass DC (2014). Firefighters and cancer: where are we and where to now?. Occup Environ Med.

[8] Kales SN, Soteriades ES, Christophi CA, Christiani DC (2007). Emergency duties and deaths from heart disease among firefighters in the United States. N Engl J Med.

[9] Gaskill S, Sharkey B, Lieberg E (2008). Health risks in incident management teams. Wildland Firefighter Health Saf Rep.

[10] Walton SM, Conrad KM, Furner SE, Samo DG (2003). Cause, type, and workers' compensation costs of injury to fire fighters. Am J Ind Med.

[11] Semmens EO, Domitrovich J, Conway K, Noonan CW (2016). A cross-sectional survey of occupational history as a wildland firefighter and health. Am J Ind Med.

[12] Britton C, Lynch CF, Ramirez M, Torner J, Buresh C, Peek-Asa C (2013). Epidemiology of injuries to wildland firefighters. Am J Emerg Med.

[13] Vincent GE, Aisbett B, Hall SJ, Ferguson SA (2016). Fighting fire and fatigue: sleep quantity and quality during multi-day wildfire suppression. Ergonomics.

[14] Hansman H (2017). A quiet rise in wildland-firefighter suicides. The Atlantic.

[15] Clement S, Schauman O, Graham T, Maggioni F, Evans-Lacko S, Bezborodovs N, Morgan C, Rüsch N, Brown JSL, Thornicroft G (2015). What is the impact of mental health-related stigma on help-seeking? A systematic review of quantitative and qualitative studies. Psychol Med.

[16] (2022). Investing in more robust mental health support for wildland firefighters. US Department of the Interior.

[17] Everyone goes home® in the wildland. Everyone Goes Home®.

[18] (2023). National Institute of Occupational Safety and Health Total Worker Health® program. US Centers for Disease Control and Prevention.

[19] Kuehl KS, Elliot DL, Goldberg L, MacKinnon DP, Vila BJ, Smith J, Miočević M, O'Rourke HP, Valente MJ, DeFrancesco C, Sleigh A, McGinnis W (2014). The safety and health improvement: enhancing law enforcement departments study: feasibility and findings. Front Public Health.

[20] Kuehl KS, Elliot DL, MacKinnon DP, O'Rourke HP, DeFrancesco C, Miočević M, Valente M, Sleigh A, Garg B, McGinnis W, Kuehl H (2016). The SHIELD (Safety and Health Improvement: Enhancing Law Enforcement Departments) study: mixed methods longitudinal findings. J Occup Environ Med.

[21] Elliot DL, Goldberg L, Duncan TE, Kuehl KS, Moe EL, Breger RKR, DeFrancesco CL, Ernst DB, Stevens VJ (2004). The PHLAME firefighters' study: feasibility and findings. Am J Health Behav.

[22] Elliot DL, Goldberg L, Kuehl KS, Moe EL, Breger RKR, Pickering MA (2007). The PHLAME (Promoting Healthy Lifestyles: Alternative Models' Effects) firefighter study: outcomes of two models of behavior change. J Occup Environ Med.

[23] Robertson M, Henning R, Warren N, Nobrega S, Dove-Steinkamp M, Tibirica L, Bizarro A (2013). The Intervention Design and Analysis Scorecard: a planning tool for participatory design of integrated health and safety interventions in the workplace. J Occup Environ Med.

[24] Stead M, Arnott L, Dempsey E (2013). Healthy heroes, magic meals, and a visiting alien: community-led assets-based social marketing. Soc Mark Q.

[25] Ahmad MS, Talib NBA (2016). Analysis of community empowerment on projects sustainability: moderating role of sense of community. Soc Indic Res.

[26] Andajani-Sutjahjo S, Liew TCH, Smith JF, Esekielu I, Mason G, Tariu I (2018). Engaging community volunteers in participatory action research in Tāmaki community of Auckland, New Zealand. Health Promot Int.

[27] Elliot DL, Goldberg L, MacKinnon DP, Ranby KW, Kuehl KS, Moe EL (2016). Empiric validation of a process for behavior change. Transl Behav Med.

[28] Mabry L, Elliot DL, Mackinnon DP, Thoemmes F, Kuehl KS (2013). Understanding the durability of a fire department wellness program. Am J Health Behav.

[29] MacKinnon DP, Elliot DL, Thoemmes F, Kuehl KS, Moe EL, Goldberg L, Burrell GL, Ranby KW (2010). Long-term effects of a worksite health promotion program for firefighters. Am J Health Behav.

[30] Morrison D, Lichtenwald K, Tang R (2020). Extending the online focus group method using web-based conferencing to explore older adults online learning. Int J Res Method Educ.

[31] Lobe B, Morgan D, Hoffman KA (2020). Qualitative data collection in an era of social distancing. Int J Qual Methods.

[32] (2023). Kardsort.

[33] (2023). Taguette.

[34] A fire department's guide to implementing NFPA 1582. International Association of Fire Chiefs.

[35] Mabry L, Bamberger M, Rugh J, Mabry L (2012). Qualitative evaluation methods. RealWorld Evaluation: Working Under Budget, Time, Data, and Political Constraints.

[36] Polanyi M (1983). The Tacit Dimension.

[37] (2023). Articulate 360.

[38] de Winter JFC, Dodou D (2010). Five-point Likert items: t test versus Mann-Whitney-Wilcoxon (addendum added October 2012). Pract Assess Res Evaluation.

[39] Cumming G (2013). Understanding the New Statistics: Effect Sizes, Confidence Intervals, and Meta-Analysis.

[40] Cohen J (1988). Statistical Power Analysis for the Behavioral Sciences.

[41] Chen SY, Feng Z, Yi X (2017). A general introduction to adjustment for multiple comparisons. J Thorac Dis.

[42] Nayebi F, Desharnais JM, Abran A (2012). The state of the art of mobile application usability evaluation.

[43] Koopmans E, Cornish K, Fyfe TM, Bailey K, Pelletier CA (2022). Health risks and mitigation strategies from occupational exposure to wildland fire: a scoping review. J Occup Med Toxicol.

[44] Groot E, Caturay A, Khan Y, Copes R (2019). A systematic review of the health impacts of occupational exposure to wildland fires. Int J Occup Med Environ Health.

[45] Navarro K (2020). Working in smoke: wildfire impacts on the health of firefighters and outdoor workers and mitigation strategies. Clin Chest Med.

[46] Adetona O, Reinhardt TE, Domitrovich J, Broyles G, Adetona AM, Kleinman MT, Ottmar RD, Naeher LP (2016). Review of the health effects of wildland fire smoke on wildland firefighters and the public. Inhal Toxicol.

[47] McConell G, Kloot K, Hargreaves M (1996). Effect of timing of carbohydrate ingestion on endurance exercise performance. Med Sci Sports Exerc.

[48] Ruby BC, Shriver TC, Zderic TW, Sharkey BJ, Burks C, Tysk S (2002). Total energy expenditure during arduous wildfire suppression. Med Sci Sports Exerc.

[49] Joe MJ, Hatsu IE, Tefft A, Mok S, Adetona O (2022). Dietary behavior and diet interventions among structural firefighters: a narrative review. Nutrients.

[50] Donahue S, McMorrow C, Almeida AA, Feairheller DL (2022). Feasibility and perception of a diet and exercise intervention delivered via telehealth to firefighters. Int J Telerehabil.

