# Supplementary material for: A Web-Based Total Worker Health Intervention for Those Fighting Wildland Fires: Mixed Methods Development and Effectiveness Trial
Source: J Med Internet Res. 2023 Oct 25;25:e47050. doi: 10.2196/47050 (PMC10632911; doi:10.2196/47050)
Supplement: Multimedia Appendix 1 [file jmir_v25i1e47050_app1.docx]

**Multimedia Appendix 1.** FEMA WFF focus group or individual interview guide.

**Additional Instructions for WebEx video calls**

**For WebEx Calls:**

1. **Begin call:** 10 minutes prior to start time to help anyone who starts early
2. **Technical difficulties**: Allow 10 minutes to assist participants
3. **Complete informed consent and survey as outline in study protocol.**
4. **Interview**
   1. Begin recording and read the following introductory script
   2. Keep track of who is talking

Before beginning the interview OHSU researcher states:

- *Thank you for your time. The goal today is to talk for about 50 minutes about health and safety for wildland fire fighters to help us build a free program to address those needs.*
- *Participation is* ***voluntary****.*
- *Please speak freely; there are no right or wrong answers. You do not need to feel that you are speaking for anyone other than yourself. We* *are interested in each individual’s viewpoint. [For WebEx add: “Please mute your microphone when you are not speaking.”]*
- *Since this is a research study, we will audio record and take notes. Recordings and notes will be kept confidential with OHSU research staff. These responses will not be shared with your managers, coworkers, or anyone who is not part of the OHSU research staff.*
- *Information gathered with be grouped from other interviews, no individual names will be used in group summaries. OHSU researchers will review the information gathered and identify patterns that emerge. With this information, we will build the learning modules of the wildland health and safety program.*
- *Please refrain from using your last name or other full names during this interview to help maintain confidentiality*

*Your input today will help shape this program with the goal of improving the health and safety of wild land fire fighters throughout the country.*

(Turn on recorder). We will now begin the interview, we have consented all who are participating and will begin.

1. To start, let’s go around introduce ourselves and please tell us your current role and your experience with wild land fires. (Use first names only.) (Unmute and tell a participant to speak)
2. Tell us about last fire season for you.
3. What is your current training schedule? How has it changed?
   1. Weekday drills?
   2. Weekend?
   3. How often do you train? Monthly?
4. What kind of training do you do right now? How has it changed from training pre-covid?
   1. (Will be different for Forest Service, seasonal, volunteers)
   2. What type of topics are covered during this training?
5. Do you get deployed to fight wildland fires? (Continue with follow-up questions if “yes”)
   1. How often are you deployed?
   2. How long are you there when you are deployed?
   3. When you are there what does your day look like?
      1. What does this deployment entail?
      2. What are your duties?
      3. How is this different than when you are at your main station?
      4. How is your life different?
6. What experience do you have with other health/safety programs for fire fighters?
   1. What works for you? What is most impactful?
   2. What doesn’t work? What is a waste of your time?
7. What are your biggest safety concerns? Before Covid-19 and after?
   1. Do you have different concerns while you are deployed? (different than when you are at your regular station)
8. What is your biggest health promotion needs?
   1. Do you have different concerns while you are deployed? (different than when you are at your regular station)

| **Potential Topics** |
| --- |
| Hearing protection |
| Personal protective Equipment |
| Hydration/heat stress |
| Sleep and fatigue |
| Physical fitness (i.e. endurance, stamina) |
| Body weight |
| Diet/nutrition |
| Musculoskeletal health (Sprains, strains etc.) |
| Injury prevention |
| Cardiovascular disease |
| Cancer risk |
| Work/life balance |
| Mental health (i.e. emotional resilience, stress management) |
| Covid-19 |

1. How do they like to learn? (Or what holds your attention?)
   1. In the classroom? Computer based or workbook to read?
   2. Online videos or audio recordings?
   3. Group classes or working on your own
   4. Drills/Activities (interactive)?
2. What topics are important to include in a health and safety program for your team that was not already mentioned?
3. How do you balance work and family demands?
4. The IAFF has placed a big emphasis on mental, emotional health; what helps you stay emotionally resilient in this work? What coping strategies do you have?
5. What are your physical health needs?
6. Tell us about health and fitness in your Department.
   1. Do you have current programs to promote this?
   2. If so, are you utilizing these resources?
   3. What resources work best? How could they improve?
7. If you volunteer as a fire fighter (or have before), why do/did you?
8. Are there any other grants or programs you have going on currently or have completed recently that were not already mentioned?
9. In general, and based on your own experience, what do you think is needed in terms of wellness and safety training in the fire service?
   1. Of these, what do you think is the most important to address? Why?
   2. Was there anything else not mentioned previously that you would like us to address when we create a health and safety program for your team?
10. When would be the best time to do this program?
    1. Right before wildfire season?
    2. In the winter?
11. How do you envision yourself completing this program?
12. As we put this program together, would you like to be involved and have your input in on the development?

THANK YOU –

This concludes the interview (stop recording). We appreciate your participation. Do you have any questions? (Hand out copy of consent form if in person, if over video offer to send an email with the consent form to each participant or Chief) If you have any additional questions, please contact us.

**FEMA WFF Interview Notes**

**Type of interview** (circle one)**: Individual / Focus Group Participant Numbers**: ___________

**Interviewer Name:** ______________________ **Note taker name**: _______________________

**Recording Device**: _______________________ **File Location:** ___________________________

**File name:** _____________________________ **Transferred to X- drive:** __________________

**Back u-up Device:** _______________________ **File Name:** _________________

**Date:** _______ / ______ / _______ **Time:** Start _______ : ______ AM / PM

End _______ : ______ AM / PM

Total _________ minutes

**Location** (select one): **Station:** ___________________________

- **Portland Fire & Rescue** (Portland, OR)
- **Stayton Fire District** (Stayton, OR)
- **Hoodland Fire District** (Welches, OR)
- **Boise National Forest** (Boise, ID)
- **Boise Fire Department** (Boise, ID)
- **Columbus Fire Department** (Columbus, MT)
- **Red Lodge Fire Rescue** (Red Lodge, MT)
- **Horry County Fire Rescue** (Conway, SC)
